# Engineered nonlinear materials using gold nanoantenna array

**DOI:** 10.1038/s41598-017-19066-3

**Published:** 2018-01-15

**Authors:** Vladimir P. Drachev, Alexander V. Kildishev, Joshua D. Borneman, Kuo-Ping Chen, Vladimir M. Shalaev, Konstantin Yamnitskiy, Robert A. Norwood, Nasser Peyghambarian, Seth R. Marder, Lazaro A. Padilha, Scott Webster, Trenton R. Ensley, David J. Hagan, Eric W. Van Stryland

**Affiliations:** 1 Department of Physics and Advanced Materials & Manufacturing Institute, University of North Texas, Denton, Texas 76203 USA; 2 Skolkovo Institute of Science and Technology, Moscow, 143026 Russia; 3 School of Electrical and Computer Engineering and Birck Nanotechnology Center, Purdue University, West Lafayette, IN 47907 USA; 4 College of Optical Sciences, University of Arizona, Tucson, AZ 85721 USA; 5 Georgia Institute of Technology, Atlanta, GA 30332 USA; 6 CREOL, The College of Optics and Photonics, University of Central Florida, Orlando, FL 32816 USA; 7 Department of Physics, University of Central Florida, Orlando, FL 32816 USA; 8 Present Address: Univ Estadual Campinas, UNICAMP, Sao Paulo, Brazil; 9 Present Address: U.S. Army Research Laboratory, Adelphi, MD 20783 USA

## Abstract

Gold dipole nanoantennas embedded in an organic molecular film provide strong local electromagnetic fields to enhance both the nonlinear refractive index (*n*_2_) and two-photon absorption (2PA) of the molecules. An enhancement of 53× for 2PA and 140× for nonlinear refraction is observed for BDPAS (4,4′-bis(diphenylamino)stilbene) at 600 nm with only 3.7% of gold volume fraction. The complex value of the third-order susceptibility enhancement results in a sign change of *n*_2_ for the effective composite material relative to the pure BDPAS film. This complex nature of the enhancement and the tunability of the nanoantenna resonance allow for engineering the effective nonlinear response of the composite film.

## Introduction

Nanoantennas have gathered great attention due to their strong, frequency tunable, electromagnetic field enhancement^1^. These large local fields enable many applications in near-field optics, nonlinear optics^2^, and fluorescence research^3,4^. Although plasmonic nanoparticles have been employed to enhance nonlinear absorption^5,6^, two-photon absorption (2PA) enhancement in those cases was mainly detected using two-photon excited luminescence, which is not the same as obtaining observable nonlinear absorption in transmission for the composite material^7,8^. This is because transmission measurements include contributions from both the loss due to the metal nanoparticles and the enhanced absorption of the molecules. Furthermore, the field enhancement is a complex quantity, which cross-couples nonlinear absorption and refraction.

Note, that nonlinear response enhancement due to the intrinsic nonlinearity of plasmonic nanoantennas has previously been explored in some detail but still attracts great interest. These include studies on nanoparticle size-dependence of the nonlinear response^9–13^, from atomic clusters to plasmonic nanocrystals of Ag and Au^14–17^, optical Kerr-effect, inverse Faraday effect and nonlinear optical activity for metal nanoparticles and aggregates^18,19^, enhancing nonlinearity by using metasurfaces and metamaterial structures^20^, theory on figure-of-merit for nonlinear response^21^, core-shell for enhanced second-harmonic^22^, metasurface defined with e-beam lithography of α-Si:H nanorods for ultrafast spectral and polarization switching^23^.

However, the enhancement of the “external” material nonlinearity caused by nanoantennas is less well studied. This paper considers nanoantentennas as a pair of nanoparticles, which allows tunability of the plasmon resonance by changing the particle geometry and/or gap size. The nanoantenna array quality requires e-beam lithography defined fabrication and the ability to resist laser damage at high powers.

The effective third-order susceptibility of a metal dielectric composite, in the simplest case^24,25^, is a product of two complex values: the enhancement factor and the third-order susceptibility of the component materials, which are both wavelength dependent. Therefore, one can engineer an effective nonlinear material by matching the nanoantenna resonance and the wavelength dependence of the susceptibility.

In this paper we describe the use of a gold nanoantenna array to enhance both the nonlinear refractive index (*n*_2_) and 2PA of the organic dye 4,4′-bis(diphenylamino)stilbene (BDPAS), which is known as a dye with relatively large 2PA^26–28^. Experimental results show a 53× enhancement for the effective nonlinear absorption, *α*_2_, of the composite layer at 600 nm, as supported by 3D finite element method (FEM) numerical simulations. The measured effective nonlinear refractive index, *n*_2_, is enhanced by a factor of 140 relative to a BDPAS layer of the same thickness. This is achieved with only 3.7% of gold volume fraction. The numerical simulations show that only 60 nm (the nanoantenna’s height) out of 175 nm of the BDPAS layer participates in the enhancement. Thus, the realistic enhancement of both the *n*_2_ and *α*_2_ is almost 3× larger.

## Results and Discussions

**Figure 1:**
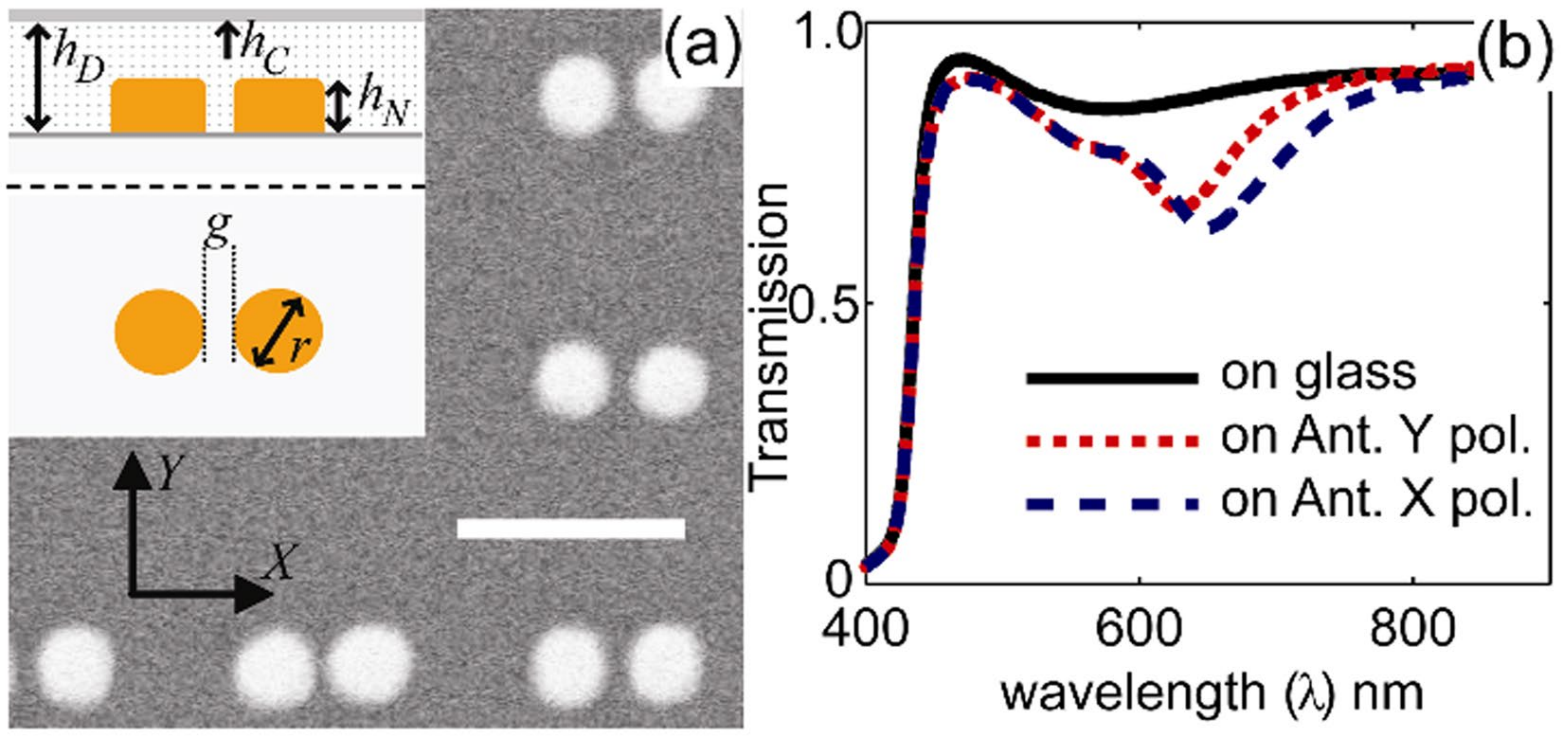
(**a**) A schematic, *h*_*D*_, *h*_*N*_, *h*_*C*_, *g*, *r* = 175, 60, 20, 14, 66 nm respectively, and SEM image of the gold nanoantenna structure.

BDPAS has a strong one-photon absorption cross-section, 2.0 × 10^−16^ cm^2^, at 390 nm^27^, and negligible linear absorption in the visible. BDPAS has 42 π-conjugated bonds and exhibits strong 2PA from 600 nm to 800 nm, with a peak 2PA cross section of *δ* = 320 GM (1 GM = 10^−50^ cm^4^s/photon) in dichloromethane (DCM) at 670 nm^27,28^. The dimensions of the gold nanoantennas shown in Fig. 1(a) (66 nm diameter, 60 nm height (including the 5 nm Ti layer), an antenna gap of 14 nm, and with an array periodicity of 250 nm) were designed so that the resonance wavelength (*λ*_*r*_) is close to the 2PA cross section peak of BDPAS.

We demonstrate 2PA enhancement using an array of gold nanoantennas on an indium-tin-oxide (ITO) coated glass substrate coated with a layer of BDPAS by the thermal evaporation in a vacuum chamber. Since the localized field enhancement caused by nanoantennas typically has a range on the order of the nanoantenna thickness, a thin BDPAS film is sufficient to take advantage of nanoantenna enhancement. Our simulations show that 60 nm thickness is enough for the geometry used. The gold nanoantennas are fabricated using electron-beam lithography, as described in the Methods section, to produce larger grain sizes and a lower loss factor, resulting in a stronger plasmonic resonance and larger local electromagnetic fields^29^. A 175 nm BDPAS film with large molecular density of *N* = 1.4 × 10^21^ cm^−3^ ^30^, is covered using the thermal evaporation method by a 20 nm layer of silica to protect the sample. The linear refractive index of the BDPAS film is approximately *n*_0_ = 1.8 at wavelengths longer than *λ* = 500 nm, as determined by spectroscopic ellipsometry. Figure 1(b) shows transmission spectra for the BDPAS film, and the film on nanoantennas at both the primary resonance polarization (X) with the incident E-field across the gap, and for the secondary polarization (Y). The transmission spectra show that the resonance wavelength, *λ*_*r*_, of these gold nanoantennas coated with a BDPAS film is 665 nm for X polarization and 630 nm for Y polarization.

**Figure 2:**
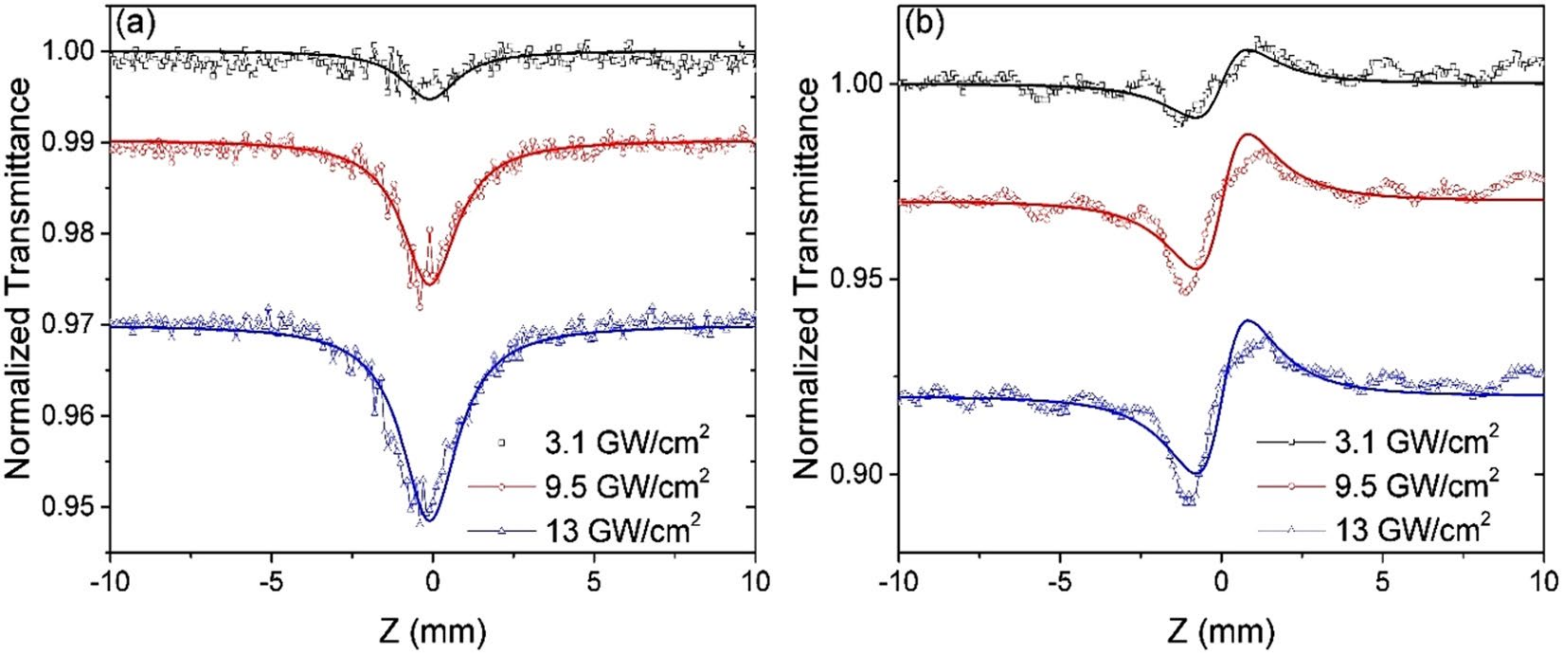
Z-scan data for 175 nm BDPAS film on nanoantennas sample [linear transmission offset for clarity]. (**a**) open-aperture with *α*_2N_(600) = 300 cm/GW fit, implying no nonlinear reflection, at 1.4, 4.3, and 6 nJ, and (**b**) Closed-aperture normalized by 1 mm glass, with *n*_2N_(600) = (0.7 ± 0.2) × 10^−11^ cm^2^/W fits, at 1.4, 4.3, and 6 nJ.

Open- and closed-aperture Z-scan measurements were used to measure the film’s nonlinear index of refraction (*n*_2_) and the nonlinear absorption coefficient (*α*_2_) for the primary X polarization (see Methods section). Introduction of the nanoantennas in the BDPAS film results in a noticeable contribution of the nonlinear reflection in the nonlinear transmission. We show below that the effect of reflection can be taken into account using the *n*_2_ obtained from the closed-aperture Z-scan in transmission. Relative to the usual method^31^, we take into account transmission through the interfaces of the nonlinear slab to retrieve *α*_2_ from the experimental results shown in Fig. 2a. Note that interface transmission does not change the wave phase, thus *n*_2_ is retrieved in the usual way^31^. The BDPAS film and film on nanoantennas (indicated herein using subscripts *F* and *N* respectively) were measured at 600 nm and 650 nm. The strong nanoantenna resonance near 650 nm caused damage at an energy per pulse smaller than ~1 nJ (2 GW/cm^2^) and did not provide usable data. Increasing the thickness of the top silica layer might be sufficient to increase the damage threshold of the nanoantennas, but further experiments are necessary to verify this.

Closed-aperture Z-scans at 600 and 700 nm, normalized by open-aperture scans, were used to measure the *n*_2_ of an 11 mM solution of BDPAS in DCM in a 1 mm cuvette at 40 nJ (41GW/cm^2^), resulting in (*n*_2(BDPAS_ _+_ _DCM)_ (600) = 4.5 ± 0.6 × 10^−16^ cm^2^/W) and (*n*_2(DCM)_ (600) = 7.0 ± 1.0 × 10^−16^ cm^2^/W). By normalizing the closed-aperture data for BDPAS in DCM by the data for pure DCM, we calculate (*n*_2(BDPAS)_ (600) = − 2.4 ± 0.8 × 10^−16^ cm^2^/W) and *(n*_2(BDPAS)_ (700) = 2.2 ± 0.8 × 10^−16^ cm^2^/W) for 11 mM of BDPAS. Note that we assume the *n*_2_ of DCM to be dispersionless across this wavelength range. This results in refractive cross-sections (*δ*_r_ = *n*_2_*ħωk*/*N*) given in units of Göppert-Mayer^32^ of *δ*_r(BDPAS)_ (600) =  −1.3 ± 0.4 × 10^2^GM and *δ*_r(BDPAS)_ (700) = 1.2 ± 0.4 × 10^2^ GM.

The *n*_2_ at 600 nm for a sample including both the BDPAS film on nanoantennas and 1 mm glass substrate is retrieved using peak to valley transmission:

1 $${\rm{\Delta }}{T}_{p-v}=0.406{(1-0.4)}^{0.25}\frac{2\pi }{\lambda \sqrt{2}}{I}_{0}({n}_{2{\rm{glass}}}{h}_{{\rm{glass}}}+{n}_{{\rm{2N}}}{h}_{{\rm{N}}}\frac{1-\exp (-{\alpha }_{{\rm{N}}}{h}_{{\rm{N}}})}{{\alpha }_{{\rm{N}}}{h}_{{\rm{N}}}})$$

From Fig. 2b Δ*T*_*p*−*v*_ = 0.0375 at 9.5 GW/cm^2^, an effective nanoantenna length of *h*_*N*_ ⋅ 0.83 and *n*_2(glass)_ = 3.0 × 10^−16^ cm^2^/W^33^. Thus we obtain an *n*_2_ for the 175 nm BDPAS film with nanoantennas layer of *n*_2N_ = (0.7 ± 0.2) × 10^−11^       cm^2^/W, see Fig. 2(b), resulting in *δ*_rN_(600) = 1.8 × 10^4^GM.

There was no detectable transmission change for the BDPAS film without nanoantennas.

Based on the known molecular density^30^, and the refractive cross-section for BDPAS, *δ*_r (BDPAS)_ (600), measured above, we give the nonlinear index of refraction of the film as *n*_2F_(600) ≈ −5.0 ± 1.4 × 10^−14^ cm^2^/W and *n*_2F_(700) ≈ 4.5 ± 1.4 × 10^−14^ cm^2^/W.

Using open-aperture Z-scans, shown in Fig. 2(a), we retrieve the nonlinear imaginary refractive index and consequently nonlinear absorption coefficient. The effect of reflection is taken into account using *n*_2_ obtained from the closed-aperture Z-scan in transmission. Since the interface transmission does not change the wave phase, *n*_2_ is retrieved in the usual way^31^. Relative to the usual method^31^, the transmission through the interfaces of the nonlinear slab is taken into account to retrieve *α*_2_ from the experimental results shown in Fig. 2a.

For intensity dependent transmission we take into account the reduced intensity at the second interface. Namely *T* = *T*_1_(*I*)*A*(*I*_eff_)*T*_2_(*Ie*^−*αh*^), where transmission at the first interface, transmission through the slab, and transmission at the second interface are: *T*_1,2_ = 1 − *R*_1,2_ = 4*n*_1,3_*n*/[(*n*_1,3_ + *n*)^2^ + *κ*^2^], *A* = *e*^−*αh*^, *n* + *iκ* = *n*_*slab*_, *α* = 4*πκ*/*λ*. The nonlinear change in transmission, taking into account the averaging coefficient 2^3/2^, is given by (see supplementary information for details):

2 $$\begin{array}{c}{2}^{3/2}\frac{{\rm{\Delta }}T}{T\,\,{\rm{\Delta }}I}=-\frac{1}{n}\frac{({n}^{2}-{n}_{1}^{2}-{\kappa }^{2})}{{({n}_{1}+n)}^{2}+{\kappa }^{2}}{n}_{{\rm{nl}}}\,+\frac{n{({n}_{1}/n+1)}^{2}+{\kappa }^{2}/n-2\kappa }{{({n}_{1}+n)}^{2}+{\kappa }^{2}}{\kappa }_{{\rm{nl}}}-\\ -\frac{4\pi h}{\lambda }{\kappa }_{{\rm{nl}}}\frac{1-{e}^{-\alpha h}}{\alpha h}-\frac{1}{n}\frac{({n}^{2}-{n}_{3}^{2}-{\kappa }^{2})}{{({n}_{3}+n)}^{2}+{\kappa }^{2}}{n}_{{\rm{nl}}}{e}^{-\alpha h}+\frac{n{({n}_{3}/n+1)}^{2}+{\kappa }^{2}/n-2\kappa }{{({n}_{3}+n)}^{2}+{\kappa }^{2}}{\kappa }_{{\rm{nl}}}{e}^{-\alpha h}.\end{array}$$

Using *n*_nl_ = *n*_2N_ = 0.7 × 10^−11^ cm^2^/W, *n*_1_ = 1.8, *n*_3_ = 1.5, from the linear reflection and transmission fitting *n* = 2.3 + *n*_nl_*I*, *k* = 0.117 + *κ*_nl_*I* at *h* = 175 nm, and ${T}_{\exp }\approx 0.6$, Δ*T*/*T* = 0.022 at Δ*I* = 13 GW/cm^2^, we obtain *κ*_2N_ = *κ*_nl_ = 0.17 × 10^−11^cm^2^/W and *α*_2N_ = *α*_nl_ = 360cm/GW.

**Figure 3:**
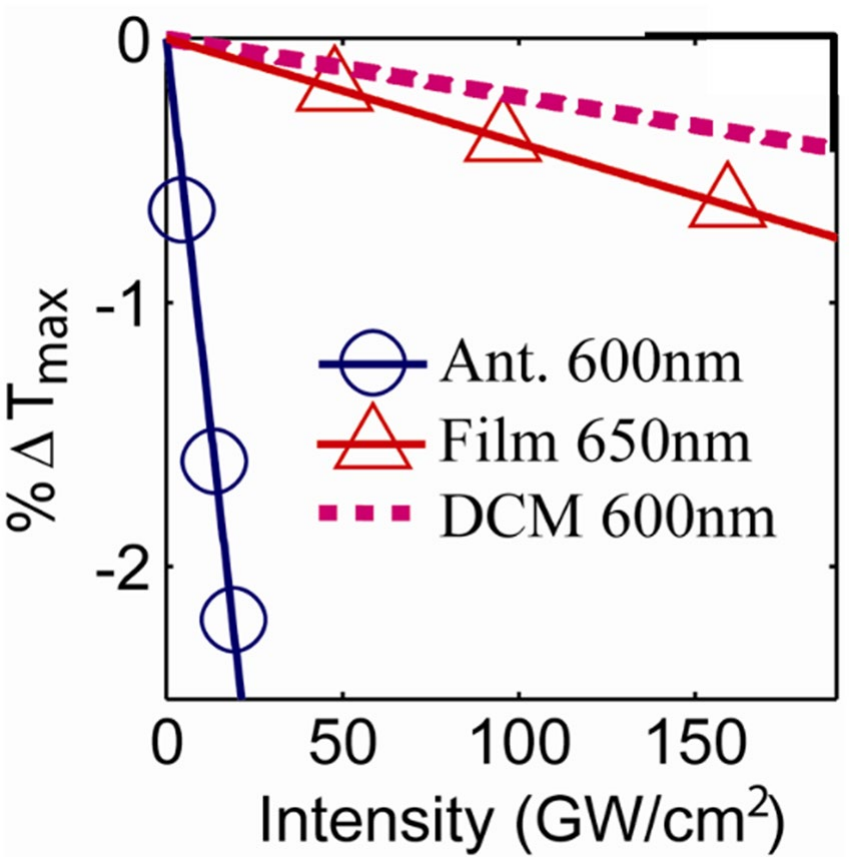
%Δ*T* from Z-scans vs. incident intensity for BDPAS with nanoantennas, BDPAS film, and BDPAS solution with exponential fit.

Note that, taking into account both absorption and reflection in the open-aperture transmission in Fig. 2a, the analysis gives *α*_2N_ = 360 cm/GW instead of *α*_2N_ = 300 cm/GW as given by the usual fitting procedure. Figure 3 shows the transmittance change (Δ*T*) versus irradiance retrieved from the open-aperture Z-scan results. Note that the nonlinear coefficients are calculated for an effective composite layer of BDPAS and nanoantennas. In the obtained results, the thickness of the effective composite layer is taken as the total thickness of BDPAS and nanoantennas. Another reasonable approach is to consider the effective layer thickness equal to the nanoantennas thickness, 60 nm, since the field enhancement is strong near the nanoparticles.

**Figure 4:**
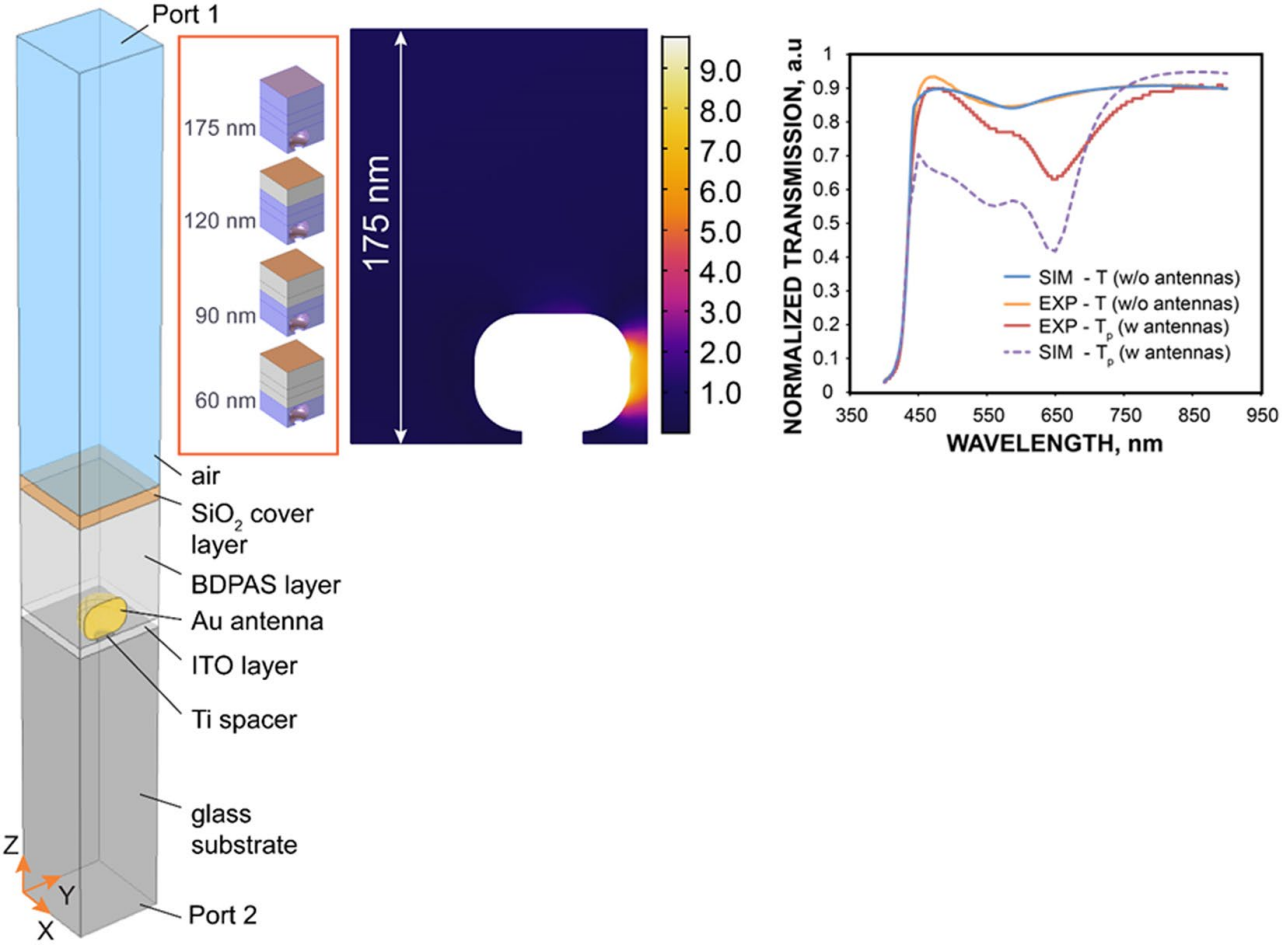
Unit cell of the FEM simulations (left), a distribution of the intensity in arbitrary units (center) at 600 nm, normalized transmission spectra with and without Au nanoantennas from the experiments and simulations (right). Simulations for BDPAS film use Eq. 3.

**Figure 5:**
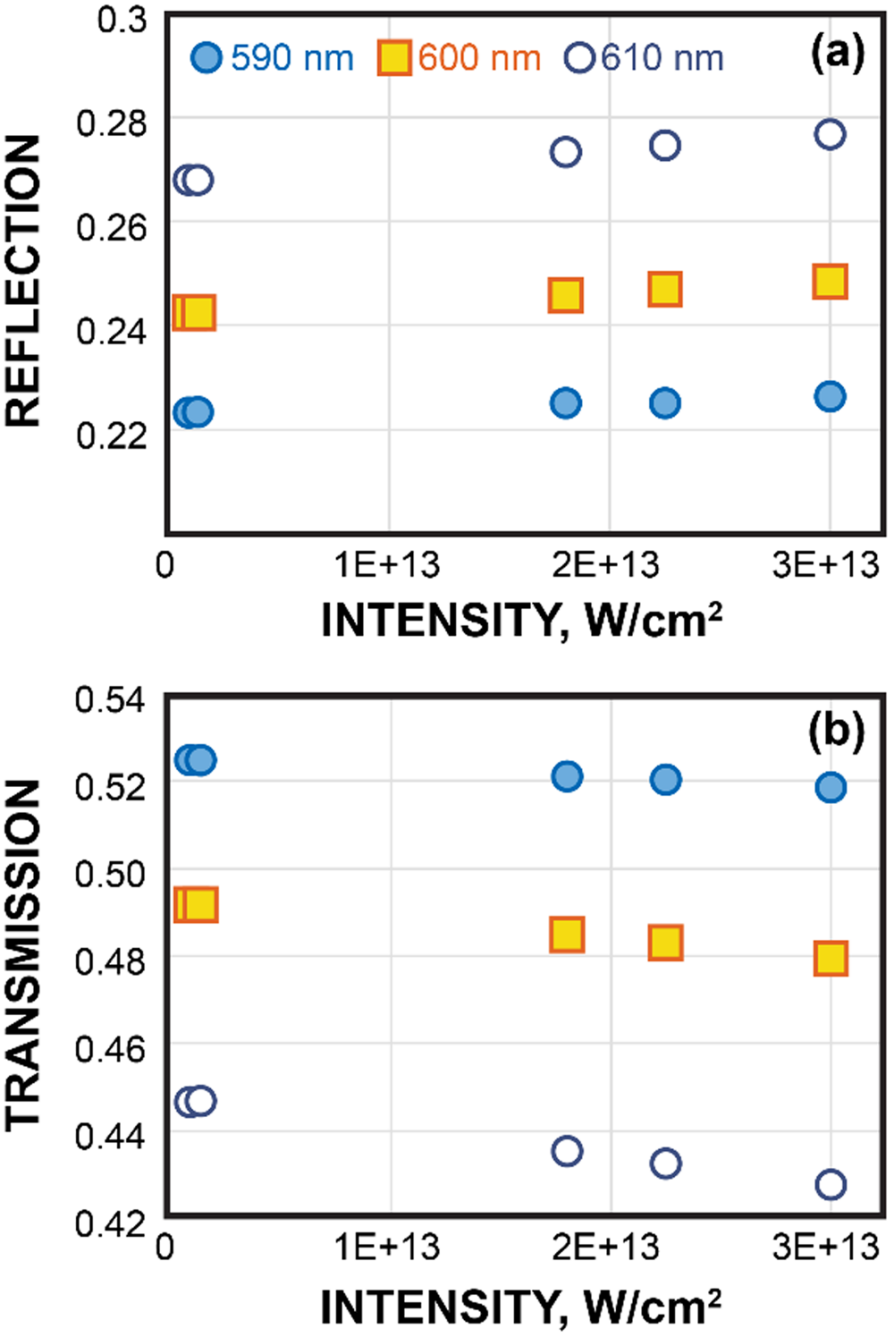
Intensity dependence of the reflection (**a**) and transmission (**b**) of the BDPAS with nanoantennas at wavelength 590, 600, and 610 nm.

**Figure 6:**
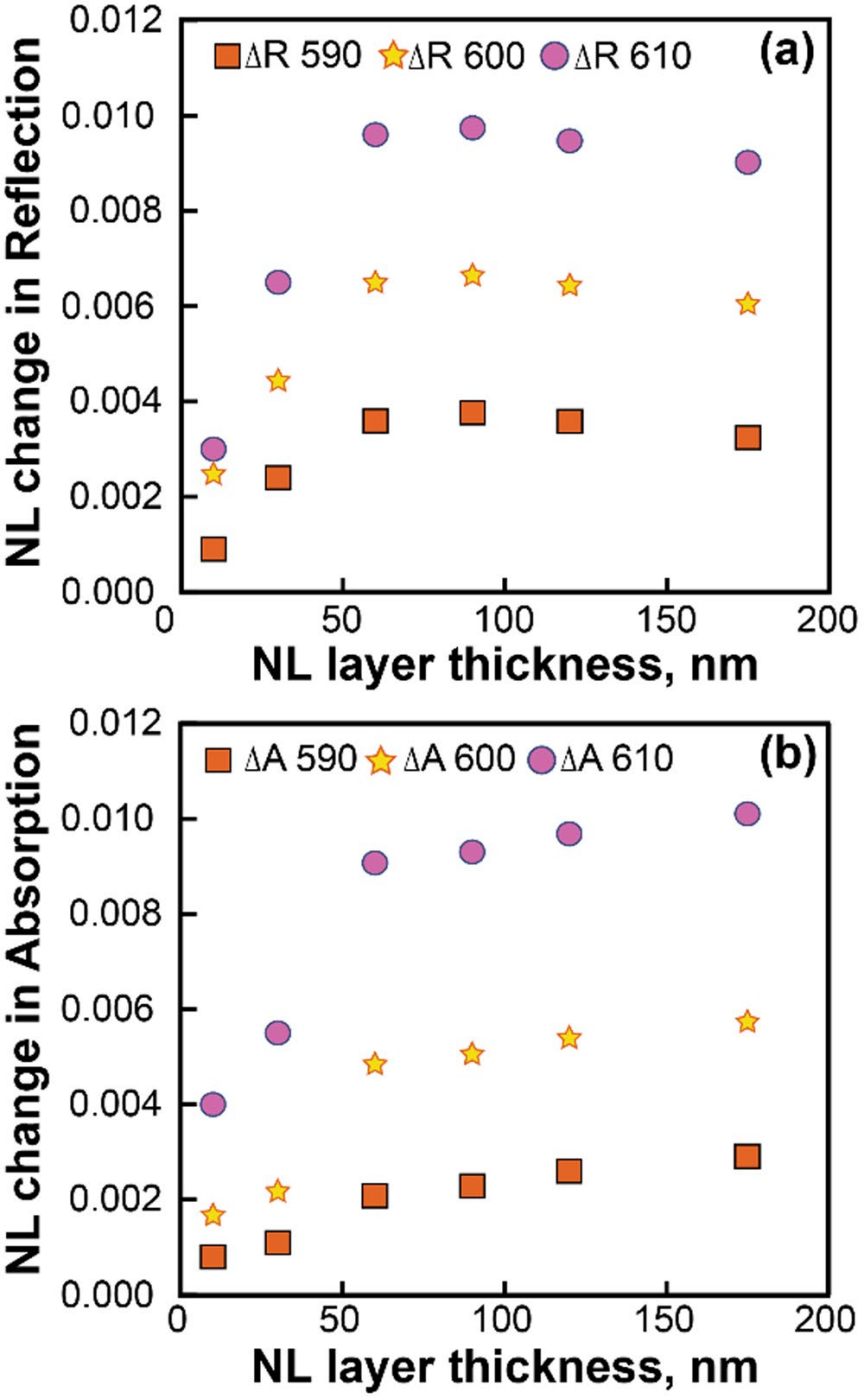
Change in absorption and reflection versus nonlinear layer (BDPAS) thickness with nanoantennas at wavelength 590, 600, and 610 nm.

Our simulations below confirm that this second approach, with an effective thickness of 60 nm, better describes the experimental situation. Indeed, the intensity mapping in Fig. 4 shows that a layer of 60 nm contains the most intense local field. Also, using the simulated intensity-dependent transmission and reflection shown in Fig. 5, we find that nonlinear changes in transmission and reflection do not grow significantly with the thickness after 60 nm, see Fig. 6. Specifically, in this case, *n*_nl_ = *n*_2N_ = 2.48 × 10^−11^ cm^2^/W, *n*_1_ = 1.8, *n*_3_ = 1.5, from linear reflection and transmission fitting *n* = 2.4 + *n*_nl_*I*, *k* = 0.31 + *κ*_nl_*I* at h = 60 nm, and we obtain *κ*_2N_ = *κ*_nl_ = 0.6 × 10^−11^ cm^2^/W and *α*_2N_ = *α*_nl_ = 1257 cm/GW.

We obtain *α*_2F_(650) = 13 cm/GW for the BDPAS film, and *α*_2N_(600) = 360 cm/GW for the BDPAS film with nanoantennas. The smaller 2PA of BDPAS at 600 nm (*δ* ≈ 130 GM in DCM) did not provide detectable attenuation to measure *α*_2F_(600 nm).

From the measured *α*_2F_(650), the 2PA cross-section of BDPAS in film form is calculated to be *δ*_F_(650) = 287 GM with ± 15% experimental error, which is in good agreement with that of BDPAS in DCM solution. Using the wavelength dependence from ref.^27^ results in an estimated *δ*_F_(600) = 162 GM, and using *δ* = (*hc*/*λ*) (*α*_2_/*N*) we obtain *α*_2F_(600 nm) = 6.8 cm/GW for the 175 nm BDPAS film. Using $\mathrm{Re}[{\chi }^{(3)}]={n}_{2}\cdot (4/3){n}_{0}^{2}{\varepsilon }_{0}c$ and $\text{Im}{\chi }^{(3)}={\alpha }_{2}\cdot (2{n}_{0}^{2}{\varepsilon }_{0}{c}^{2}/3\omega )$, we obtain_,_ ${\chi }_{N}^{(3)}(600)=(8.1+i1.9)\times {10}^{-18}{{\rm{m}}}^{{\rm{2}}}{/V}^{{\rm{2}}}$ for BDPAS on the nanoantennas, and ${\chi }_{{\rm{F}}}^{(3)}(600)=(-5.8+i3.6)\times {10}^{-20}{{\rm{m}}}^{{\rm{2}}}{/V}^{{\rm{2}}}$ and ${\chi }_{{\rm{F}}}^{(3)}(700)=(5.3+i12.7)\times {10}^{-20}{{\rm{m}}}^{{\rm{2}}}{/V}^{{\rm{2}}}$ for the BDPAS film.

The analysis of matching FEM electromagnetic simulations of the nanoantenna-dye unit-cell from Fig. 1(a) supports the experiments as described below. The geometric dimensions and Au loss factor were fit to the experimental results using an efficient simplified model described in ref.^34^. The experimental geometry was simulated with a commercial full wave electromagnetic simulation software (Comsol Multiphysics) built on the finite-element method. The one-quarter unit cell of the sample bounded by the symmetry planes is shown in Fig. 4 (left). The BDPAS layer was composed of two sublayers, nonlinear and linear. The total thickness is always 175 nm, while the nonlinear one was changed as 10, 30, 60, 90, 120, and 175 nm to find an optimal thickness of the nonlinear material coating. The intensity distribution in Fig. 4 (center) shows that the maximum nonlinear change of the refractive index is expected in the gap in correspondence with the local field distribution and the BDPAS nonlinear refractive index. Figure 4 (right) illustrates the very good correspondence of the experimental and simulated spectra of the BDPAS and a relatively good shape correspondence of the BDPAS with Au nanoantennas. The discrepancy may be due to defects in the array fabrication (missing particles in nanoantennas).

The BDPAS linear permittivity was modeled by a Tauc-Lorentz term and two Lorentz oscillators,

3 $${\varepsilon }_{{\rm{BDPAS}}}={\varepsilon }_{{\rm{TL}}}+\sum _{j=1}^{2}{\varepsilon }_{L,j}\cdot $$

A complete description of the Lorentz and Tauc-Lorentz terms along with their experimentally fitted coefficients are shown in the Supporting Information.

The refractive index inside a nonlinear host is,

4 $$n={n}_{0}+({n}_{2}+i\frac{{\alpha }_{2}}{2{k}_{0}})I,$$

where *k*_0_ = 2*π*/*λ* is a free-space wave number, and inside a NL medium the intensities and the field magnitudes are linked as,

5 $$I=\frac{1}{2}{\varepsilon }_{0}{n^{\prime} }_{0}c{|E|}^{2},\quad \quad E(t)=\mathrm{Re}E\exp (-i\omega t),$$

with *n*′_0_ being the real part of the linear component of refractive index.

In the FEM model, the nonlinear properties of the bulk film BDPAS have been taken into account by using *n*_2_ = −5.0 × 10^−18^ m^2^/W and *α*_2_ = 6.7 × 10^−11^ m/W, while the real and imaginary parts of *χ*^(3)^ were computed as

6 $$\mathrm{Re}[{\chi }^{(3)}]=\frac{4}{3}{\varepsilon }_{0}cn{^{\prime} }_{0}^{2}{n}_{2}$$

7 $$\text{Im}[{\chi }^{(3)}]=\frac{2}{3}{\varepsilon }_{0}c{n^{\prime} }_{0}^{2}{\alpha }_{2}/{k}_{0}$$

with the principal components of the anisotropic dielectric function in the medium defined as,

8 $${\varepsilon }_{ii}={n}_{0}^{2}+\frac{3}{4}{\chi }_{ii}^{(3)}{|{E}_{i}|}^{2}i\in \{x,y,z\}$$

The solution of resulting nonlinear wave equation $\nabla \times \nabla \times {\rm{E}}-{k}_{0}\,\varepsilon \,{\rm{E}}=0$ in the entire domain is obtained self-consistently at each excitation step with the commercial FEM solver (COMSOL Multiphysics, Wave Optics Module) using a high-performance iterative method (double dogleg). We should note that to avoid possible numerical issues and speed up simulations the illumination intensity is taken to be gradually increasing with the previous solution used as a seed for a new step.

From the literature^35,36^ for “fast” nonlinearity we take ${\chi }_{{\rm{Au}}}^{(3)}=(-1.1+i1.0)\times {10}^{-22}\,{{\rm{m}}}^{2}/{{\rm{V}}}^{2}\ll {\chi }_{{\rm{BDPAS}}}^{(3)}$. The simulated intensity dependent transmission and reflection coefficients are shown in Fig. 5.

The thickness dependence (Fig. 6) shows that the nonlinear response is almost saturated after 60 nm. The nonlinear transmission and reflection obtained with the full wave simulations were then fitted with the variable complex refractive index *N* = *n* + *ik* mimicking the intensity dependence. This is done with the J. A. Wollam software^37,38^. All the layers are glass 1 mm (*n* = 1.52) without backside reflection, ITO 15 nm (*n* = 1.9586), BDPAS 175 nm, SiO_2_ 20 nm (*n* = 1.458). The fitted refractive index corresponds to the BDPAS parameters without nanoantennas and to the effective nonlinear refractive index and effective nonlinear absorption of the effective uniform layer in case of the BDPAS with nanoantennas. Thus for 175 nm BDPAS film we get at 600 nm:

$$n=1.8-16\times {10}^{-4}(I/{I}_{{\rm{nl}}}),\,k=0.00537+9.3\times {10}^{-4}(I/{I}_{{\rm{nl}}}),\,{\rm{where}}{I}_{{\rm{nl}}}=3\times {10}^{14}\,\frac{W}{{m}^{2}}.$$

This retrieval procedure via the fitting gives for the BDPAS *n*_2F_(600) = −16 × 10^−4^/*I*_nl_ = −5.3 × 10^−18^ m^2^/W, *κ*_2_ = 9.3 × 10^−4^/*I*_nl_ = 3.1 × 10^−18^, and *α*_2F_(600) = 2*k*_0_*κ*_2_ = 6.5 × 10^−11^ m/W. This provides a control retrieval result matching the accepted starting parameters of the BDPAS layer.

The BDPAS with Au nanoantennas and effective layer thickness L = 175 nm at 600 nm results in: *n* = 2.3 + 0.018(*I*/*I*_nl_), *k* = 0.117 + 0.0033(*I*/*I*_nl_), where *I*_nl_ = 2.9 × 10^13^ *W*/*m*^2^. Thus at 175 nm for the effective uniform layer *n*_2eff_(600) = 0.62 × 10^−11^ cm^2^/W, *α*_2eff_(600) = 240 cm/GW. Experimental results give *n*_2N_ = (0.7 ± 0.2) × 10^−11^ cm^2^/W, *α*_2N_ = 360 cm/GW.

Since approximately only the first 60 nm near the nanoantennas contribute to the enhancement (see Fig. 6), it makes sense to retrieve the complex nonlinear refractive index for the composition of 60 nm of the effective nonlinear BDPAS/Au nanoantennas hybrid and keep the remaining 115 nm as a linear layer. The retrieved effective refractive index for the 60 nm uniform effective layer is: *n* = 2.4 + 0.021(*I*/*I*_*nl*_), *k* = 0.31 + 0.015(*I*/*I*_*nl*_),where *I*_nl_ = 2.9 × 10^13^ *W*/*m*^2^. Correspondingly, this gives for the 60 nm effective layer *n*_2eff_(600) = 0.7 × 10^−11^ cm^2^/W,*α*_2eff_(600) = 1100 cm/GW.

We also evaluate another approach to define an effective third-order susceptibility, ${\chi }_{{\rm{eff}}}^{(3)}$, widely used in the earlier literature^25^ and recent developments^5^. Namely ${\chi }_{{\rm{eff}}}^{(3)}$ is defined as Eq. 9, where *f*, *g*^(3)^ are the volume filling fraction and enhancement factor respectively, subscripts {h, in} denote the host (dye) and inclusions (gold).

9 $${X}_{{\rm{eff}}}^{(3)}={f}_{{\rm{in}}}{g}_{{\rm{in}}}^{(3)}{\chi }_{{\rm{in}}}^{(3)}+{f}_{{\rm{h}}}{g}_{{\rm{h}}}^{(3)}{\chi }_{{\rm{h}}}^{(3)}$$

The E-field extracted from simulations was used to find the complex nonlinear enhancement $({g}_{{\rm{h}}}^{(3)})$, *f*_h_ = 0.964 from the above geometry since ${X}_{{\rm{eff}}}^{(3)}$ in our system is dominated by the host (BDPAS) term. One should mention that the approximation based on the local field factor is not practical as this follows from our evaluation in the supplementary information. We conclude that local field enhancement is strongly intensity dependent and cannot be used to define the effective nonlinear susceptibilities. Any detectable nonlinearity will make the approximation used in the literature^5,25^ unacceptable.

Table 1 summarizes the experimental and simulations’ results. The final enhancement of the nonlinear susceptibility of the dye due to the nanoantennas is found by comparing both the experimental results (${\chi }_{{\rm{N}}}^{(3)}$) and the FEM simulations (${X}_{{\rm{eff}}}^{(3)}$) of the composite structure to the BDPAS film (${\chi }_{{\rm{F}}}^{(3)}$).

**Table 1:** Summary of experimental and simulation results.

	${{\bf{n}}}_{{\bf{2}}}({\bf{600}}),\times {\bf{1}}{{\bf{0}}}^{{\boldsymbol{-}}{\bf{18}}}{{\bf{m}}}^{{\bf{2}}}/{\bf{W}}$	${{\boldsymbol{\alpha }}}_{{\bf{2}}}({\bf{600}}),\times {{\bf{10}}}^{-{\bf{11}}}{\bf{m}}/{\bf{W}}$	${{\boldsymbol{\chi }}}^{({\bf{3}})}({\bf{600}}),\times {{\bf{10}}}^{-{\bf{20}}}{{\bf{m}}}^{{\bf{2}}}/{{\bf{V}}}^{{\bf{2}}}$	$\frac{{{\boldsymbol{n}}}_{{\bf{2N}}}}{{{\boldsymbol{n}}}_{{\bf{2F}}}}$	$\frac{{{\boldsymbol{\alpha }}}_{{\bf{2N}}}}{{{\boldsymbol{\alpha }}}_{{\bf{2F}}}}$
BDPAS Film	−5	6.8	−5.8 + *i*3.6		
BDPAS with NAs Exp (175 nm)	700	360	812 + *i*190	−140	53
BDPAS with NAs Sim (175 nm)	620	240	720 + *i*37	−125	36
BDPAS with NAs Sim (60 nm + 115 nm)	700	1100	810 + *i*580	−140	160

The nonlinear refractive index of the BDPAS layer is enhanced by *n*_2N_/*n*_2F_ = −140x from experiment, and *n*_2Neff_/*n*_2F_ = −125x from simulations at 600 nm. The retrieval using the effective thickness 60 nm, which corresponds to the characteristic thickness of the nonlinear response saturation presented in Fig. 6, gives *n*_2Neff_/*n*_2F_ = −140. While the sign change in *n*_2_ is reproduced in both cases, the magnitude deviation is most likely due to the difference in the resonance quality between the experimental sample and simulated unit cell, which is seen for the linear response in Fig. 1b. The 2PA of the dye layer is enhanced by *α*_2N_/*α*_2F_ = 53 times in the experiment, and *α*_2Neff_/*α*_2F_ = 36 times from simulations at 600 nm. Note that the enhancement due to the nanoantennas changes the sign of the nonlinear refractive index. Such a complex enhancement of the local field in a composite medium has been discussed earlier^39^. The main reason for the imaginary part of the local field enhancement is the phase shift between the local field and applied field. It is also important that the local field varies in both, magnitude and phase with coordinates near the nanoantenna particles. The simulations suggest also that the nonlinear changes in transmission have a contribution from both nonlinear changes in reflection and in absorption. However, the Z-scan data for *n*_2_ allows separating the effects of absorption to get *α*_2_ as was shown above.

## Conclusions

Gold dipole nanoantennas that produce strong local electromagnetic fields were used to enhance both the nonlinear refractive index and 2PA of a nonlinear dye. Bi-periodic gold nanoantenna arrays with nonlinear dyes also have a large wavelength dependent theoretical enhancement as has been shown from simulations. Furthermore, experimental results have shown that the nonlinear refractive index is enhanced by 140× with a sign change from negative to positive and a 53× enhancement in nonlinear (2PA) absorption at *λ* = 600 nm for BDPAS (4,4′-bis(diphenylamino)stilbene) with only 10% of surface coverage or 3.7% volume fraction of the gold nanoantennas. Since only 60 nm out of 175 nm of the BDPAS layer contributes to the enhancement, the realistic enhancement of both the *n*_2_ and *α*_2_ is ~3 times larger.

Both real and imaginary parts of the BDPAS ${\chi }_{{\rm{F}}}^{(3)}$ contribute to the effective nonlinear absorption of the BDPAS/Au nanoantenna composite material, due to the complex nature of the enhancement factor resulting in the sign flip for the nonlinear refractive index. This fact, along with the tunable enhancement of the plasmon resonance, makes it possible to control the effective nonlinear response of the composite film. The above results demonstrate that the nonlinear composite can be further optimized through appropriate selection of component materials.

## Methods

The gold nanoantennas are fabricated using electron-beam lithography on an ITO-coated glass substrate with a 15 nm ITO layer. An electron-beam evaporator is used to produce a 5 nm adhesion layer of titanium followed by a 55 nm gold film with a 1 Å/s deposition rate in a 7 × 10^−7^ Torr vacuum chamber (Airco). After lift-off the nanoantennas are annealed at 400 °C for 2 minutes by a rapid thermal processing system (RTP, Minipulse RTA), which has been shown to produce larger grain sizes and a lower loss factor, resulting in a stronger plasmonic resonance and larger local electromagnetic fields^29^. A 175 nm BDPAS film was then deposited using thermal evaporation in a vacuum chamber, followed by a 20 nm layer of silica to protect the sample. The linear refractive index of the BDPAS film is determined by spectroscopic ellipsometry, which is approximately *n*_0_ = 1.8 at wavelengths longer than *λ* = 500 nm. Open- and closed-aperture Z-scan measurements were used to measure the film’s nonlinear index of refraction (*n*_2_) and the nonlinear absorption coefficient (*α*_2_) for the primary X polarization. The effect of reflection can be taken into account using the *n*_2_ obtained from the closed-aperture Z-scan in transmission. Relative to the usual method^31^, we take into account transmission through the interfaces of the nonlinear slab to retrieve *α*_2_ from the experimental results as explained in supplementary information. Note that interface transmission does not change the wave phase, thus *n*_2_ is retrieved in the usual way^31^. A femtosecond optical parametric amplifier, OPA (Light Conversion, TOPAS-800), pumped by a Ti:Sapphire regenerative amplifier laser system (Clark MXR, CPA 2010), with a pulse duration of 140 fs (FHWM), 1 kHz repetition rate, was used. The focused spot size varied for different measurements, so both pulse energy and irradiance are provided.

## Electronic supplementary material

Supplementary Information
