# Supplementary material for: Engineered nonlinear materials using gold nanoantenna array
Source: Sci Rep. 2018 Jan 15;8:780. doi: 10.1038/s41598-017-19066-3 (PMC5768724; doi:10.1038/s41598-017-19066-3)
Supplement: Supplementary file 1 — Supplementary Information [file 41598_2017_19066_MOESM1_ESM.pdf]

Supplementary Information

Engineered nonlinear materials using gold nanoantenna array

Vladimir. P. Drachev[1,†*], Alexander V. Kildishev[2], Joshua D. Borneman[2], Kuo-Ping Chen[2], Vladimir M. Shalaev[2], Konstantin Yamnitskiy,[3] Robert A. Norwood[3], N. Peyghambarian[3], Seth R. Marder[4], Lazaro A. Padilha[5,6], Scott Webster[5], Trenton R. Ensley[5,7], David J. Hagan[5,8], and Eric W. Van Stryland[5,8]

[1]*Department of Physics and Advanced Materials&Manufacturing Institute, University of North Texas, Denton, Texas 76203, USA*

[†]*Skolkovo Institute of Science and Technology, Moscow 143026, Russia*

[2]*School of Electrical and Computer Engineering and Birck Nanotechnology Center, Purdue University, West Lafayette, IN 47907, USA*

[3]*College of Optical Sciences, University of Arizona, Tucson, AZ 85721, USA*

[4]*Gergia Institute of Technology, Atlanta, GA 30332, USA*

[5]*CREOL, The College of Optics and Photonics, University of Central Florida, Orlando, FL 32816, USA*

[6]*Current Address: Univ Estadual Campinas, UNICAMP, Sao Paulo, Brazil*

[7]*Current Address: U.S. Army Research Laboratory, Adelphi, MD 20783, USA*

[8]*Department of Physics, University of Central Florida, Orlando, FL 32816, USA*

**Corresponding author: vladimir.drachev@unt.edu*

Section 1. Derivation of the Equation (2).

We show, that using open-aperture Z-scans, one can retrieve the nonlinear absorption coefficient without measurements Z-scan in reflection. For intensity dependent transmission we take into account reduced intensity at the second interface due to reflection. Namely

$$T = T_1(I)A(I_{\mathrm{eff}})T_2(Ie^{-\alpha h}), \tag{S1.1}$$

where transmission at the first interface, transmission through the slab, and transmission at the second interface are:

$$T_{1,2} = 1 - R_{1,2} = 4n_{1,3}n \Big/ \left[\left(n_{1,3}+n\right)^2 + \kappa^2\right],\ \ A = e^{-\alpha h}, n + i\kappa = n_2, \alpha = 4\pi\kappa/\lambda . \tag{S1.2}$$

Nonlinear change in the transmission, taken into account the averaging coefficient $2^{3/2}$:

$$2^{3/2}\Delta T = AT_2\frac{dT_1}{dI}\Delta I + AT_1\frac{dT_2}{dI}\Delta I e^{-\alpha h} + T_1T_2\frac{dA}{dI}\Delta I_{eff},$$
$$\text{where } \Delta I_{eff} = \Delta I\frac{1-e^{-\alpha h}}{\alpha h}. \tag{S1.3}$$

We assume that only the second layer has an appreciable imaginary part and nonlinearity.

$$\frac{dT_i}{dI} = \frac{\partial T_i}{\partial n}n_{\mathrm{nl}} + \frac{\partial T_i}{\partial \kappa}\kappa_{\mathrm{nl}} \ \text{ and } \ \frac{dA}{dI} = \frac{dA}{d\kappa}\kappa_{\mathrm{nl}} . \tag{S1.4}$$

Thus:

$$2^{3/2}\frac{\Delta T}{T\,\Delta I} = -\frac{1}{n}\frac{\left(n^2-n_1^2-\kappa^2\right)}{\left(n_1+n\right)^2+\kappa^2}n_{\mathrm{nl}} + \frac{n\left(n_1/n+1\right)^2+\kappa^2/n-2\kappa}{\left(n_1+n\right)^2+\kappa^2}\kappa_{\mathrm{nl}} -$$
$$-\frac{4\pi h}{\lambda}\kappa_{\mathrm{nl}}\frac{1-e^{-\alpha h}}{\alpha h} - \frac{1}{n}\frac{\left(n^2-n_3^2-\kappa^2\right)}{\left(n_3+n\right)^2+\kappa^2}n_{\mathrm{nl}}e^{-\alpha h} + \frac{n\left(n_3/n+1\right)^2+\kappa^2/n-2\kappa}{\left(n_3+n\right)^2+\kappa^2}\kappa_{\mathrm{nl}}e^{-\alpha h}. \tag{S1.5}$$

Section 2. Dielectric function of BDPAS

For the photon energy (ω) in eV the BDPAS linear permittivity $\varepsilon_{\mathrm{BDPAS}}(\omega)$ is modeled as a sum,

$$\varepsilon_{\mathrm{BDPAS}}(\omega) = \varepsilon_{\mathrm{TL}}(\omega) + \sum_{j=1}^{2} \varepsilon_{\mathrm{L},j}(\omega), \tag{S2.1}$$

of a Tauc-Lorentz term ($\varepsilon_{\mathrm{TL}}(\omega)$, see (S2) and (S3)) and two Lorentz terms, $\varepsilon_{\mathrm{L},j}(\omega) = a_j b_j \omega_{0,j} \big/ \left(\omega_{0,j}^2 - \omega^2 - i b_j \omega\right)$.

The real part of the Tauc-Lorentz term, $\mathrm{Re}(\varepsilon_{\mathrm{TL}})$, is derived from the imaginary part of $\varepsilon_{\mathrm{TL}}(\omega)$,

$$\mathrm{Im}(\varepsilon_{\mathrm{TL}}) = \begin{cases} \dfrac{\omega_0}{\omega} \dfrac{a_0 c \left(\omega - \omega_{\mathrm{g}}\right)^2}{\left(\omega_0^2 - \omega^2\right)^2 + c^2 \omega^2}, & \omega > \omega_{\mathrm{g}}; \\ 0, & \omega \le \omega_{\mathrm{g}}. \end{cases} \tag{S2.2}$$

using the Kramers-Kronig integration following Jelisson and Modine [S1]:

$$\begin{aligned} \mathrm{Re}(\varepsilon_{\mathrm{TL}}) = {} & \varepsilon_\infty + \frac{a_0 c}{\pi \zeta^4(\omega)} \frac{a_{\mathrm{ln}}(\omega)}{2\alpha\omega_0} \ln\left[\frac{\omega_0^2 + \omega_g^2 + \alpha\omega_g}{\omega_0^2 + \omega_g^2 - \alpha\omega_g}\right] \\ & - \frac{a_0}{\pi\zeta^4(\omega)} \frac{a_{\mathrm{atan}}(\omega)}{\omega_0} \left[\pi - \arctan\left(\frac{\alpha + 2\omega_g}{c}\right) + \arctan\left(\frac{\alpha - 2\omega_g}{c}\right)\right] \\ & + 2\frac{a_0\omega_0}{\pi\alpha\zeta^4(\omega)} \omega_{\mathrm{g}} \left(\omega^2 - \gamma^2\right) \left[\pi + 2\arctan\left(2\frac{\gamma^2 - \omega_{\mathrm{g}}^2}{\alpha c}\right)\right] \\ & - \frac{a\omega_0 c}{\pi\zeta^4(\omega)} \frac{\omega^2 - \omega_{\mathrm{g}}^2}{\omega} \ln\left(\frac{\left|\omega - \omega_{\mathrm{g}}\right|}{\omega + \omega_{\mathrm{g}}}\right) + 2\frac{a_0\omega_0 c}{\pi\zeta^4(\omega)} \omega_{\mathrm{g}} \ln\left[\frac{\left|\omega - \omega_{\mathrm{g}}\right|\left(\omega + \omega_{\mathrm{g}}\right)}{\sqrt{\left(\omega^2 - \omega_{\mathrm{g}}^2\right)^2 + \omega_g^2 c^2}}\right] \end{aligned}, \tag{S2.3}$$

with the auxiliary functions and constants being defined as $\zeta^4(\omega) = \left(\omega^2 - \gamma^2\right)^2 + \alpha^2 c^2/4$, $a_{\mathrm{ln}}(\omega) = \left(\omega_{\mathrm{g}}^2 - \omega^2\right)\omega^2 + \omega_{\mathrm{g}}^2 c^2 - \omega_0^2\left(\omega_0^2 + 3\omega_{\mathrm{g}}^2\right)$, $a_{\mathrm{atan}}(\omega) = \left(\omega^2 - \omega_0^2\right)\left(\omega_0^2 + \omega_{\mathrm{g}}^2\right) + \omega_{\mathrm{g}}^2 c^2$, $\alpha = \sqrt{4\omega_0^2 - c^2}$, $\gamma = \sqrt{\omega_0^2 - c^2/2}$.

Table S1. The fitted coefficients of the Tauc-Lorentz material model.

Terms	Coefficients	Comments
Tauc-Lorentz Term, $\varepsilon_{\mathrm{TL}}(\omega)$	$a_0 = 358.5 \pm 3.5$	
	$\omega_0 = 2.79 \pm 0.00083;\ \mathrm{eV}$	
	$\omega_g = 0.28762 \pm 0.0065\,\mathrm{eV}$	
	$c_{.1} = 2.9 \pm 1000\,\mathrm{eV}$	This constant is singular - fitting is insensitive to this parameter
Lorentz Terms, $\varepsilon_{\mathrm{L},j}(\omega),\ j = \{1, 2\}$	$a_1 = 0.0554 \pm 0.004;$	$j = 1$
	$b_1 = 0.49165 \pm 0.034\ \mathrm{eV}$	
	$\omega_{01} = 2.1074 \pm 0.0058\ \mathrm{eV}$	
	$a_2 = 761.93 \pm 1.13\mathrm{E}+003$	$j = \{1, 2\}$
	$b_2 = 0.0162 \pm 0.015\ \mathrm{eV}$	
	$\omega_{02} = 0.16 \pm 0.33\ \mathrm{eV}$	

The coefficients of Table S1 are fitted using the experimental transmission spectra with the J.A.Wollam Co. software [S2] and material model (S1) achieving with the mean squared error, MSE = 0.8426. MSE=$\sqrt{\chi^2/(2N-M)}$, where N is the number of fitted points including spectral, M is the number of fitting variable parameters of the model, χ^2 is the chi-square likelihood estimator. The fitting quality is seen in Fig.4 (right).

Section 3. Evaluation of $\chi_{\mathrm{eff}}^{(3)}$ using local field factor approximation

This approach to define an effective third-order susceptibility, $\chi_{\mathrm{eff}}^{(3)}$, is widely used in the literature [5,10]. Namely $\chi_{\mathrm{eff}}^{(3)}$ is defined as Eq. S3.1, S3.2, where f, $g^{(3)}$ are the volume filling fraction and enhancement factor respectively, subscripts $\{\mathrm{h}, \mathrm{in}\}$ denote the host (dye) and inclusions (gold), $\langle\ \rangle_{\mathrm{V}}$ is an average over the volume, $\vec{E}^2 = \vec{E}\cdot\vec{E}$ (complex), and $|\vec{E}|^2 = \vec{E}^H \cdot \vec{E}$ (real).

$$X_{\mathrm{eff}}^{(3)} = f_{\mathrm{in}} g_{\mathrm{in}}^{(3)} \chi_{\mathrm{in}}^{(3)} + f_{\mathrm{h}} g_{\mathrm{h}}^{(3)} \chi_{\mathrm{h}}^{(3)} \tag{S3.1}$$

$$g_j^{(3)} = \left\langle \vec{E}^2 \left|\vec{E}\right|^2 \right\rangle_{\mathrm{V}_j} \Big/ \left(\langle \vec{E} \rangle_{\mathrm{V}}^2 \left| \langle \vec{E} \rangle_{\mathrm{V}} \right|^2 \right),\ j = \{\mathrm{h}, \mathrm{in}\}. \tag{S3.2}$$

here $\left\langle \vec{E} \right\rangle_{\mathrm{V}} = \frac{1}{V}\int_V \vec{E}dv$, $\left\langle \vec{E} \right\rangle_{\mathrm{V}}^2 = \left\langle \vec{E} \right\rangle_{\mathrm{V}} \cdot \left\langle \vec{E} \right\rangle_{\mathrm{V}}$, $\left|\langle \vec{E} \rangle_{\mathrm{V}}\right|^2 = \left\langle \vec{E} \right\rangle_{\mathrm{V}} \cdot \left\langle \vec{E} \right\rangle_{\mathrm{V}}^*$, and

$\left\langle \vec{E}^2 \left|\vec{E}\right|^2 \right\rangle_{\mathrm{V}_j} = \frac{1}{V_j}\int_{V_j} (\vec{E}\cdot\vec{E})(\vec{E}\cdot\vec{E}^*)dv$, $j = \{\mathrm{h}, \mathrm{in}\}$.

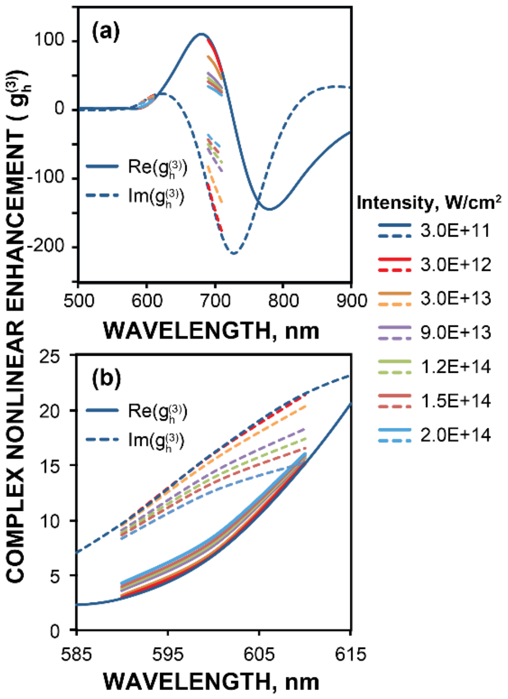

Fig. S3.1. Spectra of the $\mathrm{Re}\,g_h^{(3)}$ (solid lines) and $\mathrm{Im}\,g_h^{(3)}$ (dashed lines) calculated with Eq.(8) and simulated with FEM model for comparison at different intensities and wavelengths 590, 600, 610, 700, 710, 720 nm. a), b) correspond to the same parameters but different spectral range and vertical scale.

The E-field was extracted from simulations and (S3.2) was used to find the complex nonlinear enhancement $(g_\mathrm{h}^{(3)})$, shown in Fig.S3.1(a,b) for the primary X polarization taking $f_\mathrm{h} = 0.964$ from the above geometry. As is mentioned, $X_\mathrm{eff}^{(3)}$ in our system is dominated by the host (BDPAS) term.

One should mention that the approximation based on the local field factor is not practical as this follows from the comparison in Fig.S3.1 between nonlinear simulations and calculations based of the equations (S3.1-S3.2). It is seen that local field enhancement is strongly intensity dependent and cannot be used to define the effective nonlinear susceptibilities. Any detectable nonlinearity will make the approximation unacceptable.

References

S1. Jellison, G.E.; and Modine, F.A.; *Appl.Phys. Lett* **1996**, 69 (3), 371-374, Erratum, Jellison G.E.; and Modine, F.A.; *Appl.Phys. Lett* **1996**, 69 (14), 2137.

S2. The simulations for the experimental and the effective, stratified, planar structures were performed with ellipsometry software (W-VASE, J. A. Woollam Co., Inc.) that is based on a 4x4 scattering-matrix method for solving Maxwell’s Equations Ref. [22].
